# Supplementary material for: Phillygenin Attenuated Colon Inflammation and Improved Intestinal Mucosal Barrier in DSS-induced Colitis Mice via TLR4/Src Mediated MAPK and NF-κB Signaling Pathways
Source: Int J Mol Sci. 2023 Jan 23;24(3):2238. doi: 10.3390/ijms24032238 (PMC9917337; doi:10.3390/ijms24032238)
Supplement: Supplementary file 1 [file ijms-24-02238-s001.zip › ijms-2088234-supplementary.pdf]

Table S1 KEGG signaling pathways

KEGG signaling pathway	Count (%)	P-value	Fold Enrichment	FDR
Ras signaling pathway	16.77852349	7.99145E-13	6.243842365	2.83696E-11
PI3K-Akt signaling pathway	19.46308725	6.18811E-12	4.746731235	1.34463E-10
MAPK signaling pathway	16.77852349	1.2845E-10	4.927113703	2.02665E-09
Prolactin signaling pathway	8.724832215	1.80908E-09	10.76081633	2.10623E-08
C-type lectin receptor signaling pathway	10.06711409	2.07657E-09	8.357142857	2.10623E-08
T cell receptor signaling pathway	10.06711409	2.07657E-09	8.357142857	2.10623E-08
Relaxin signaling pathway	10.73825503	4.26989E-09	7.186710963	3.78953E-08
Estrogen signaling pathway	10.73825503	1.09082E-08	6.718012422	8.15247E-08
Rap1 signaling pathway	12.75167785	1.54495E-08	5.24244898	1.04468E-07
VEGF signaling pathway	7.382550336	4.84563E-08	10.80290557	2.77444E-07
Chemokine signaling pathway	11.40939597	1.53218E-07	5.130357143	8.05813E-07
FoxO signaling pathway	9.395973154	3.22975E-07	6.192366412	1.47943E-06
IL-17 signaling pathway	8.053691275	5.15793E-07	7.396960486	2.21947E-06
Neurotrophin signaling pathway	8.724832215	7.88941E-07	6.329891957	3.11193E-06
ErbB signaling pathway	7.382550336	1.65266E-06	7.498487395	6.17572E-06
Fc epsilon RI signaling pathway	6.711409396	2.0537E-06	8.521008403	7.47757E-06
GnRH signaling pathway	7.382550336	3.7981E-06	6.853456221	1.25426E-05
HIF-1 signaling pathway	7.382550336	1.58801E-05	5.847444299	4.50995E-05
TNF signaling pathway	6.711409396	0.000121079	5.173469388	0.000245618
Sphingolipid signaling pathway	6.711409396	0.000192633	4.869147659	0.000369647
Phospholipase D signaling pathway	7.382550336	0.000215225	4.306563707	0.000407493
Thyroid hormone signaling pathway	6.711409396	0.000218578	4.78866588	0.000408396
NOD-like receptor signaling pathway	8.053691275	0.000304149	3.778881988	0.000546914
Adipocytokine signaling pathway	4.697986577	0.001122992	5.87826087	0.001822751
p53 signaling pathway	4.697986577	0.001508082	5.556164384	0.002379418
JAK-STAT signaling pathway	6.711409396	0.001809481	3.576719577	0.002792894
NF-kappa B signaling pathway	5.369127517	0.00198811	4.457142857	0.003035608
Insulin signaling pathway	6.040268456	0.002373841	3.806465068	0.003511306
mTOR signaling pathway	6.040268456	0.005251816	3.342857143	0.007170748
Toll-like receptor signaling pathway	4.697986577	0.008738453	3.9	0.010981065
Oxytocin signaling pathway	5.369127517	0.016446368	3.010018553	0.019960549
Apelin signaling pathway	4.697986577	0.03195446	2.917985612	0.036593011

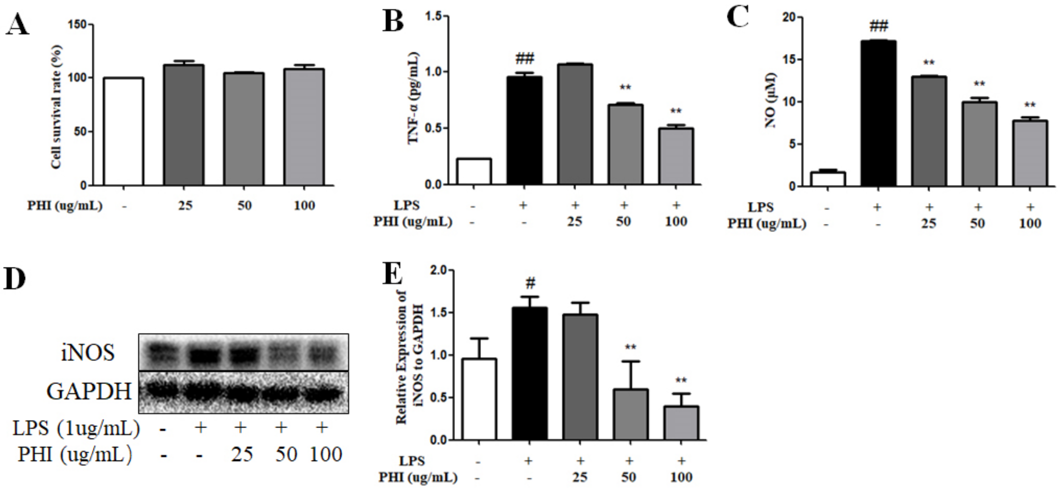

Figure S1. PHI inhibited the inflammatory response in LPS-Stimulated RAW264.7 Cells. (A) Cell survival rate (%). The content of (B) TNF-*α* and (C) NO in RAW264.7 cells was detected according to the assay kit methods. (D-E) The expression level of iNOS protein in RAW264.7 cells were detected by western blotting. $^{\#}P < 0.05$, $^{\#\#}P < 0.01$, and $^{\#\#\#}P < 0.001$ *vs*. control group. $^{*}P < 0.05$, $^{**}P < 0.01$, and $^{***}P < 0.001$ *vs*. LPS-stimulated group. ($n = 3$ per group).
